# Impact of gender in patients with continuous-flow left ventricular assist device therapy in end-stage heart failure

**DOI:** 10.1177/03913988211006715

**Published:** 2021-03-30

**Authors:** Alina Zubarevich, Marcin Szczechowicz, Anja Osswald, Arian Arjomandi Rad, Robert Vardanyan, Michel Pompeu BO Sá, Jef Van den Eynde, Bastian Schmack, Daniel Wendt, Achim Koch, Nikolaus Pizanis, Markus Kamler, Arjang Ruhparwar, Alexander Weymann, Konstantin Zhigalov

**Affiliations:** 1 Department of Thoracic and Cardiovascular Surgery, West German Heart and Vascular Center, University of Duisburg-Essen, Essen, Germany; 2 Department of Medicine, Faculty of Medicine, Imperial College London, London, UK; 3 Department of Cardiovascular Surgery at the Pronto Socorro Cardiológico de Pernambuco (PROCAPE), Recife, PE, Brazil; 4 Department of Cardiothoracic Surgery, Heart Center Essen Huttrop, University Hospital Essen, Essen, Germany; 5 Department of Cardiovascular Diseases, University Hospitals Leuven, Leuven, Belgium

**Keywords:** Gender, female gender, LVAD, heart failure, MCS

## Abstract

**Background:**

There is an ongoing debate about the influence of the female gender on postoperative outcomes after durable left ventricular assist device (LVAD) implantation. Despite the differences in pathophysiology of heart failure in females, therapy concepts are the same as in the male population. The aim of this study was to investigate the role of the female gender in surgical heart failure therapy.

**Materials and methods:**

Between August 2010 and January 2020, 207 patients were treated with durable LVAD at out institution. We matched 111 patients in two groups to compare the outcomes in male and female patients and to stratify the risk factors of mortality.

**Results:**

The groups were matched 2:1 and were comparable after matching. We found no difference in in-hospital and follow-up mortality between male and female patients. Postoperative adverse events and complications were found to be unvaried across male and female patients. Female patients had higher rates of postoperative LVAD-thrombosis compared to their male counterparts (13.5% vs 0, *p* = 0.001) and the rates of renal replacement therapy lasting over 90 days were also higher in the female group (33.8% vs 56.8%, *p* = 0.021). Furthermore, the female gender was not an independent predictor neither of in-hospital nor follow-up mortality.

**Conclusions:**

Durable continuous flow left ventricular assist devices as a bridge to transplantation or recovery in female patients are associated with a higher risk of acute kidney injury requiring RRT and are at a higher risk of LVAD-thrombosis. Nevertheless, survival rates between genders are similar.

## Introduction

**Figure 1.**
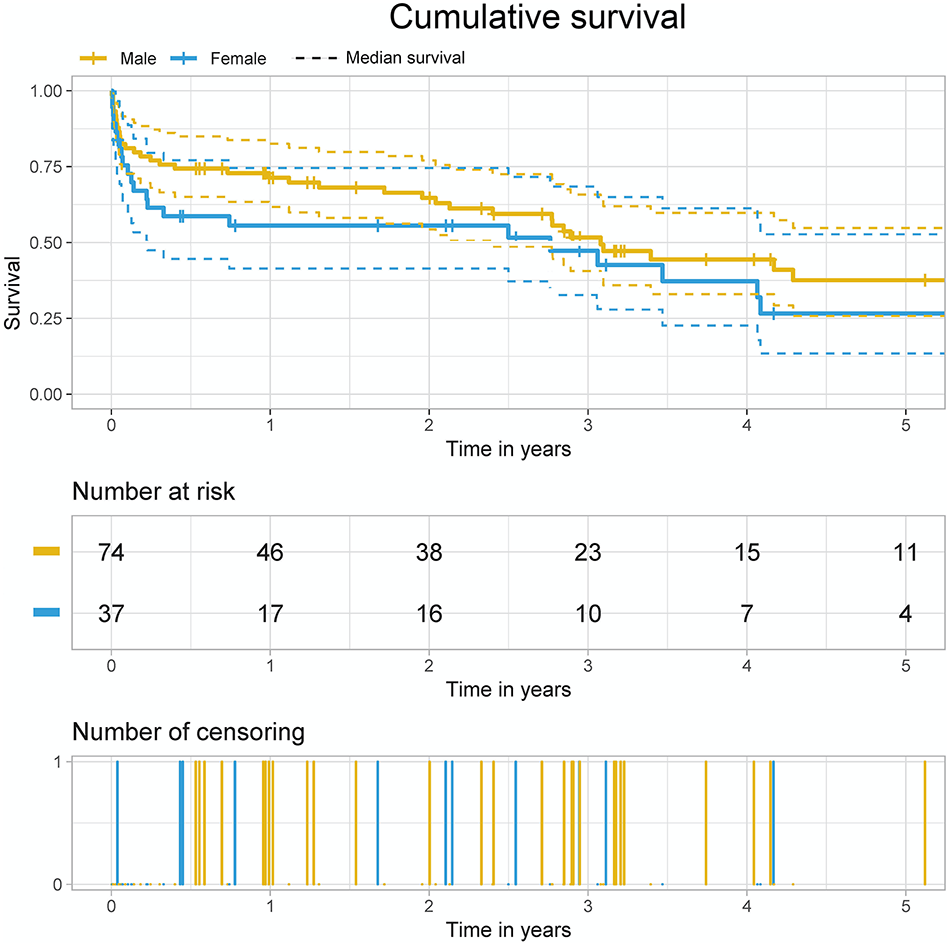
Overall survival (*p* = 0.292).

The American Heart Association’s Heart Disease and Stroke Statistics—2020 Update reports that the prevalence of heart failure (HF) is on the rise, affecting over 6.5 million in Americans above the age of 20.^1^ HF remains the leading cause of mortality and morbidity, impacting both genders equally. However, randomized control trials investigating left ventricular device (LVAD) therapy present only 20% to 25% of subjects as women, with this underrepresentation being explained by the limited thoracic space in female patients not being able to sufficiently house the relatively large earlier generation LVAD’s.^2–5^ Moreover, it has been described that the pathophysiology and subsequent epidemiology of HF in female patients differs to that of male patients.^6–9^ Nevertheless, a lack of large gender-specific clinical trials analyzing the durable mechanical circulatory support (MCS) therapy exists. In this study we sought to analyze our single center experience with patients treated using durable continuous flow LVAD’s in order to determine the impact of the variable “female gender” on postoperative outcomes. We propensity score matched female patients to male patients (Figure 1) to present gender differences in patients undergoing durable MCS therapy, thus contributing to the current body of evidence regarding LVAD implantation in female patients and gender-related post-LVAD implantation outcomes.

## Materials and methods

### Study population

At our institution, the West German Heart and Vascular Center (Essen, Germany), from August 2010 to January 2020, a total of 207 patients were treated with a durable LVAD due to different causes (Tables 1 and 3). Operation was indicated in those patients according to current guidelines at the time. Two LVAD models were implanted: HeartMate III (HM III) (Thoratec Corp., Pleasanton, CA, USA) and HeartWare (HVAD) (HeartWare International Inc., Framingham, MA, USA). The choice of VAD was based upon availability of the device in the clinic and the personal decision and preference of the surgeon. Median follow-up time was 2.0 years (IQR 0.24–3.39 years).

**Table 1.** Baseline characteristics.

Variables	Male (*n* = 74)	Female (*n* = 37)	*p* Value
Age, years	57.26 ± 11.6	57.57 ± 12.26	0.869
INTERMACS Class 1–2, *n* (%)	47 (63.5%)	25 (67.6%)	0.673
EF, %	18.0 ± 6.75	19.2 ± 8.42	0.434
Pericardial effusion	11 (14.9%)	4 (10.8%)	0.556
Pulmonary hypertension	44 (59.5%)	21 (56.8%)	0.785
Cardiovascular risk factors			
Arterial hypertension	42 (56.8%)	18 (48.6%)	0.419
Hyperlipidemia	34 (45.9%)	16 (43.2%)	0.787
Nicotin abuse	39 (52.7%)	20 (54.1%)	0.893
Family history	13 (17.6%)	10 (27.0%)	0.246
Diabetes mellitus	26 (35.1%)	11 (29.7%)	0.569
Coronary arterial disease	42 (56.8%)	17 (45.9%)	0.282
Peripheral arterial disease	12 (16.2%)	6 (16.2%)	1.0
Preoperative myocardial infarction	31 (41.9%)	15 (40.5%)	0.892
Preoperative pacemaker	23 (31.1%)	11 (29.7%)	0.884
Preoperative defibrillator implantation	40 (54.1%)	20 (54.1%)	1.0
Preoperative CRTD	27 (36.5%)	9 (24.3%)	0.197
Atrial fibrillation	23 (31.1%)	16 (43.2%)	0.206
Chronic obstructive lung disease	13 (17.6%)	5 (13.5%)	0.585
Apoplex	5 (6.8%)	5 (13.5%)	0.241
Preoperative infection	21 (28.4%)	13 (35.1%)	0.467
Mechanical ventilation	20 (27.0%)	6 (16.2%)	0.205
IABP	12 (16.2%)	6 (16.2%)	1.0
ECMO	21 (28.4%)	6 (16.2%)	0.159
Primary diagnosis, *n* (%)			
dCMP	44 (59.5%)	22 (59.5%)	1.0
iCMP	27 (36.5%)	13 (35.1%)	0.889
Toxic induced CMP	2 (2.7%)	2 (5.4%)	0.471

CMP: cardiomyopathy; CRTD: cardiac resynchronization therapy defibrillator; dCMP: dilatative cardiomyopathy; ECMO: extracorporeal membrane oxygenation; IABP: intra-aortic balloon pump; iCMP: ischemic cardiomyopathy.

### Study design

The study is a retrospective review of prospectively collected data. Data collected as part of the institutional Mechanical Circulatory Support Database included detailed information on patient demographics; baseline clinical characteristics; laboratory, echocardiographic, and hemodynamic parameters; and other intraoperative variables and postoperative outcomes. The follow-up data was collected at planned periodic presentations of patients at our VAD clinic. The study was approved by the local ethics committee.

### Study groups

Patients were stratified according to their gender (male/female) and matched with the nearest-neighbor method and ratio 1:2. Group 1 (*n* = 74) represented male patients and the Group 2 (*n* = 37) represented female patients. The matching was performed using to the following preoperative characteristics: age, body mass index, arterial hypertension, hyperlipidemia, smoking history, history of myocardial infarction, history of percutaneous coronary intervention, history of internal defibrillator implantation, peripheral arterial disease, diabetes mellitus, chronic obstructive lung disease, history of brain ischemia, any active infection, INTERMACS level, bridge to transplantation, dilatative cardiomyopathy, ischemic cardiomyopathy, toxic cardiomyopathy, serum creatinine level, ejection fraction, aortic valve stenosis, mitral valve regurgitation, tricuspid valve regurgitation, pulmonary hypertension, intraoperative use of hemadsorption filter.

### Outcome measures

We sought to determine the impact of the female gender on postoperative outcomes in patients with end-stage heart failure undergoing LVAD implantation. The primary endpoint was mortality (in-hospital and on follow-up) while the secondary endpoints were adverse events and other postoperative outcomes following LVAD implantation. Patients were censored after their death or at the cutoff of the study.

### Variables and definitions

The variables that were evaluated included baseline characteristics; preoperative clinical data; preoperative laboratory parameters; intraoperative data; postoperative variables; and follow-up data. The adverse events were defined according to “INTERMACS Adverse Event Definitions.”^10^

### Statistical analysis

To create a control group with reduced preoperative differences, we performed a propensity-score matching with the nearest-neighbor method and ratio 1:2. The standardized mean differences are presented in Figure 1. The continuous variables are presented as means ± standard deviations and compared between the groups using Student’s *t*-test when normally distributed. Otherwise, they are presented as medians with quartiles and compared with Mann-Whitney *U* Test. The normality of distributions was checked with the Kolmogorov-Smirnov Test. Categorical variables are presented as absolute numbers and percentages. Categorical variables were compared between the groups with the chi-square test if its assumptions were met, otherwise we used the Fisher’s exact test. The independent risk-factors for hospital mortality were identified with univariate logistic regression and presented as odds-ratios (OR) with 95% confidence intervals (95% CI). The independent risk-factors for long-term mortality were identified with univariate proportional hazard regression and presented as hazard-ratios (HR) with 95% confidence intervals (95% CI). We compared the survival curves with the log-rank test. We used the Kaplan–Meier method to analyze the survival. The significance of survival differences between the groups was assessed with Log-Rank and Breslow tests. A value of *p* < 0.05 was considered to be statistically significant. To perform statistical analysis, we used IBM SPSS Statistics, Version 25.0. Armonk, NY: (IBM Corp. Released 2017), and R (R Core Team, 2017).

## Results

### Baseline characteristics

The mean age at surgery was 59 (51–65) and male/female ratio was 170/37 (17.9% were female). In this study we sought to compare the postoperative outcomes in female and male patients. Therefore, we matched the patients in two groups. After matching, a total of 111 patients (the male group included 74 patients and the female group 37 patients) underwent a gender-adjusted analysis. The patients’ age, indication for the LVAD implantation, and INTERMACS class were not significantly different between the groups. We also found no significant differences in patients’ comorbidities (Table 1) and preoperative blood-work findings.

### Intraoperative characteristics

Intraoperatively, there was no significant differences between the two groups in terms of indications and therapy strategy, operating time, CPB-time, use of hemadsorption, concomitant procedures, use of cardioplegia and intraoperative implantation of additional MCS (Table 2).

**Table 2.** Intraoperative parameters.

Variables	Male (*n* = 74)	Female (*n* = 37)	*p*-Value
Indications			
Destination therapy	51 (68.9%)	27 (73.0%)	0.66
Bridge-to-transplant	18 (24.3%)	9 (24.3%)	1.0
Bridge-to-candidacy	5 (6.8%)	1 (2.7%)	0.373
Operating time, min	219.35 ± 72.96	216.14 ± 89.64	0.84
CPB-time, min	88.16 ± 38.65	89.37 ± 33.45	0.87
Cytosorb	27 (36.5%)	11 (29.7%)	0.479
Device:			
HeartWare LVAD	66 (89.2%)	34 (91.9%)	0.653
HM III LVAD	8 (10.8%)	3 (8.1%)	0.653
Isolated procedure	60 (81.1%)	34 (91.9%)	0.136
Concomitant procedure	14 (18.9%)	3 (8.1%)	0.136
AVR	6 (8.1%)	1 (2.7%)	0.269
CABG	1 (1.4%)	0	0.478
TVR	5 (6.8%)	1 (2.7%)	0.373
ASD-closure	3 (4.1%)	0	0.214
LAA-exclusion	5 (6.8%)	1 (2.7%)	0.373
VSD-closure	2 (2.7%)	1 (2.7%)	1.0
LV-Aneurysma	1 (1.4%)	0	0.478
Cardioplegia	8 (10.8%)	1 (2.7%)	0.14
ST-RVAD (intraoperative)	4 (5.4%)	2 (5.4%)	1.0
VV-ECLS (intraoperative)	0	1 (2.7)	0.155

AVR: aortic valve replacement; ASD: atrial septal defect; CABG: coronary artery bypass surgery; HM III LVAD: heart mate III left ventricular assist device; LAA: left atrial appendage; LV: left ventricle; ST-RVAD: right ventricular assist device; TVR: tricuspid valve repair; VSD: ventricular septal defect; VV-ECLS: veno-venous extracorporeal life support.

**Table 3.** Short-term outcomes (in-hospital).

Variables	Male (*n* = 74)	Female (*n* = 37)	*p*-Value
VV-ECLS (postoperative)	2 (2.7%)	4 (10.8%)	0.075
ST-RVAD (postoperative)	4 (5.4%)	2 (5.4%)	1.0
Apoplex	4 (5.4%)	2 (5.4%)	1.0
Intracranial hemorrhage	2 (2.7%)	4 (10.8%)	0.075
Hypoxic encephalipathia	2 (2.7%)	1 (2.7%)	1.0
Neurologic complications	7 (9.5%)	4 (10.8%)	0.822
Re-exploration for bleeding	17 (23.0%)	4 (10.8%)	0.123
LVAD-thrombosis	0	5 (13.5%)	0.001
Hemolysis	1 (1.4%)	0	0.478
Hepatic dysfunction	7 (9.5%)	5 (13.5%)	0.517
Inotropes >7d	35 (47.3%)	19 (51.4%)	0.678
Initropes >14d	21 (28.4%)	16 (43.2%)	0.117
Mild RHF	15 (20.3%)	6 (16.2%)	0.607
Moderate RHF	17 (23.0%)	12 (32.4%)	0.285
Severe RHF	9 (12.1%)	6 (16.2%)	0.556
Dialysis >90d	25 (33.8%)	21 (56.8%)	0.021
Mechanical ventilation >144 h	33 (44.6%)	15 (40.5%)	0.684
Re-intubation	8 (10.8%)	9 (24.3%)	0.062
Trachostomia	18 (24.3%)	7 (18.9%)	0.52
Respiratory failure	33 (44.6%)	17 (45.9%)	0.893
Sternal wound infection	10 (13.5%)	4 (10.8%)	0.686
Driveline-infection	3 (4.1%)	0	0.214
Postoperative pneumonia	16 (21.6%)	7 (18.9%)	0.741
Postop sepsis	12 (16.2%)	10 (27.0%)	0.178
Major infection	29 (39.2%)	16 (43.2%)	0.682
In-hospital death	15 (20.3%)	12 (32.4%)	0.159
Follow-up death	39 (52.7%)	22 (59.5%)	0.5
Number still on LVAD	32 (43.2%)	13 (35.2%)	0.412
LVAD-exchange	2 (2.7%)	1 (2.7%)	1.0
Heart transplantation	2 (2.7%)	2 (5.4%)	0.471
Recovery from LVAD	0	1 (2.7%)	0.155
Cause of death			
Cardiopulmonary failure	10 (13.5%)	4 (10.8%)	0.686
Multiorgan failure	15 (20.3%)	10 (27.0%)	0.422
Bleeding	0	1 (2.7%)	0.155
Infection	12 (16.2%)	8 (21.6%)	0.485
Cerebrovasc accident	11 (14.9%)	7 (18.9%)	0.585
unknown	6 (8.1%)	2 (5.4%)	0.604

### Survival data and adverse events

There were no significant differences between the two groups (male vs female) regarding 30-day, 1-year, and 3-year survival (log rank [Mantel-Cox] *p* = 0.292; Breslow [Generalized Wilcoxon] *p* = 0.201): 82% versus 75%; 70% versus 54% and 51% versus 47% respectively (Figure 1). Whereas there were no significant differences in the occurrence of postoperative complications such as stroke, re-exploration for bleeding, right heart failure, and major infection, female patients had a significantly higher rate of acute kidney failure receiving dialysis longer than 90 days (*p* = 0.021). Furthermore, a higher rate of LVAD thrombosis (*p* = 0.001) and of intracranial hemorrhage (2.7% vs 10.8%, *p* = 0.075) was found amongst the female cohort, albeit the statistical significance has not been fully achieved (Tables 3 and 4). On performing univariate analysis to determine the independent risk factors of the in-hospital and the follow-up mortality, it was discovered that the female gender had no impact on mortality in our cohort (logistic regression *p* = 0.162; Cox regression *p* = 0.294) (Table 5). Operating time, CPB-time, INTERMACS Class, preoperative creatinine and bilirubin levels, urea, CRP, LI-6 and PCT were all predictors of mortality in our cohort. No effect on mortality in the use of different types of LVAD devices was noted.

**Table 4.** Outcomes during follow-up.

Variables	Male (*n* = 74)	Female (*n* = 37)	*p*-Value
Postoperative apoplex	10 (13.5%)	3 (8.1%)	0.404
Intracranial bleeding	11 (14.9%)	8 (21.6%)	0.373
Hypoxic encephalopathia	5 (6.8%)	0	0.106
Overall neurological complications	21 (28.4%)	9 (24.3%)	0.650
Follow up thoracal bleeding	11 (14.9%)	2 (5.4%)	0.144
GI-bleeding	13 (17.6%)	8 (21.6%)	0.607
LVAD-thrombosis	10 (13.5%)	10 (27.0%)	0.081
Driveline infection	17 (23.0%)	12 (32.4%)	0.285
Device malfunction	2 (2.7%)	1 (2.7%)	1.0
Right heart failure during follow-up	13 (17.0%)	5 (13.5%)	0.585

**Table 5.** Independent risk factors of in-hospital mortality—univariate logistic regression.

Characteristics	Odds ratio with 95% confidence interval	*p*-Value
Age, years	1.03 (0.990–.073)	0.144
BSA	0.568 (0.093–3.46)	0.539
BMI	1.023 (0.934–1.121)	0.62
Preop. WBC	1.059 (0.946–1.185)	0.319
Preop. CRP	1.162 (1.063–1.270)	0.001
Preop. creatinine	1.934 (0.932–4.013)	0.077
Preop. urea	20.783 (2.717–158.962)	0.003
Preop. blood urea nitrogen	663.193 (8.5–51,723.92)	0.003
Preop. bilirubin	1.904 (1.254–2.889)	0.002
Preop. ALT	0.999 (0.998–1.001)	0.5
Preop. LDH	1.0 (0.999–1.001)	0.758
Preop. IL-6	1.016 (1.001–1.030)	0.039
Preop. PCT	1.576 (1.027–2.418)	0.037
Preop. EF	1.015 (0.952–1.082)	0.654
Operating time	1.008 (1.002–1.013)	0.008
CPB time	1.013 (1.001–1.025)	0.041
Intermacs level	0.585 (0.370–0.925)	0.022
Perpheral arterial disease	1.714 (0.574–5.119)	0.334
COLD	0.870 (0.260–2.907)	0.820
Diabetes	1.534 (0.63–3.76)	0.35
Arterial hypertension	1.62 (0.67–3.95)	0.288
AS	0.887 (0.092–8.570)	0.918
AR	0.470 (0.123–1.806)	0.272
TR	1.229 (0.449–3.362)	0.688
Preop. pericardial effusion	1.154 (0.335–3.975)	0.82
Female gender	1.888 (0.774–4.605)	0.162
HM III LVAD	0.285 (0.035–2.333)	0.242
TV-concomitant procedure	3.375 (0.639–17.819)	0.152

AR: aortic regurgitation; AS: aortic stenosis; BMI: body mass index; BSA: body surface area; TR: tricuspid regurgitation; TV: tricuspid valve.

## Discussion

Heart failure is an important cause of morbidity and mortality in women, with a significantly later onset than male individuals.^8^ Furthermore, various studies have demonstrated superior outcomes in male patients over female patients after multiple cardiac procedures.^11^ Due to the specifics of female HF pathophysiology, women tend to preserve their left ventricular function longer than men, therefore achieving the end-stage of the disease at an older age. When presenting with end-stage HF, women are more likely to describe severe symptomatology, but their prognosis is as poor as in male patients.^4^ Even though, the incidence of HF in female patients does not differ from the one in the male population, female patients remain strongly underrepresented in the HF clinical trials.^12,13^ There is an ongoing debate concerning gender differences in surgical end-stage HF therapy. Due to the underrepresentation of female patients and the absence of specific clinical trials on women presenting with end-stage HF, the existing results are altogether controversial and inconclusive. A further aspect to consider with regards to the underrepresentation of women in such studies is the less aggressive treatment of female patients compared to their male counterparts. Indeed, although the incidence of HF has declined over the last 50 years, female and elderly patients have not experienced a similar prognosis improvement to the male population.^14,15^

Our study assessed in-hospital and follow-up mortality of 210 patients who underwent durable LVAD implantation at our center. 111 patients were matched 2:1 in two groups comparing the outcomes between male and female patients, seeking to determine the impact of female gender on the postoperative course and outcomes. Our results illustrated no significant difference in both in-hospital and follow-up mortality (average follow-up period of 2.2 years) between male and female patients. The findings of the following analyses are supported by the literature, which also reports no difference in 30-day, 1-year, and 3-year survival between male and female patients treated with durable LVADs.^2,16,17^

In their work Coyle et al. presented the results of the MOMENTUM 3 trial showing that patients supported with a HeartMate III had significantly less pump thrombosis and major infection compared to patients with HeartWare (at 2 years). Although there were no differences in survival, functional status the HeartWare LVAD was associated with a higher morbidity compared to the HM III. In our cohort 10.8% of male patients and 8.1% of their female counterparts were treated with HM III LVAD device and the rest (the vast majority) underwent a HeartWare LVAD implantation. So, the differences between studies could be partially be explained by the different devices used, but even than unfortunately the survival rates remain controversial.

Nayak et al. conducted a statistical analysis using the International Society for Heart and Lung Transplantation Database to examine the influence of gender on the outcomes after LVAD implantation. This study demonstrated that female gender is associated with higher mortality after LVAD implantation, but this effect is observed only in the first 4 months after the assist device implantation.^18,19^ The authors claim that this phenomenon is driven by worsening of the right ventricular dysfunction and the LVAD-patient size mismatch. In our study, we did not see a significant worsening of the right ventricular dysfunction in the female group compared to the matched male group. The size mismatch issue is a general problem in LVAD therapy in female patients, as the thoracic volume within this population (much like as in pediatric patients) makes it often nearly impossible to house the LVAD-device due to its size.^20^ On the other hand, Zafar et al.^21^ in their study on patients with a body surface area of <1.5 m^2^ treated with LVAD showed no evidence of lower survival rates due to LVAD-patient size mismatch. In our cohort, the body surface area was not significantly different between male and female patients. Therefore, we didn’t have the mismatch problem specifically in the female arm. Moreover, the majority of female patients (91.9%) underwent a HeartWare device implantation, which has a smaller profile as HM III device and is easier to be housed in the female chest cavity. Postoperative adverse events and complications, including neurological complications, right heart failure, respiratory complications, device malfunction, infection, and hepatic dysfunction (Table 4) were found to be unvaried across male and female patients in our cohort. Nevertheless, we discovered a significantly increased rate of LVAD-thrombosis during the in-hospital period in the female group. Indeed, our findings are supported by the studies conducted by Lopilato et al.^22^ and Yin et al.^23^ which demonstrated increased rates of pump thrombosis in female patients. We examined the independent predictive factors of in-hospital and follow-up morbidity and mortality and discovered in the univariate analysis that female gender did not predict mortality in our cohort. The latter could be considered debatable as prior studies exist showing contradictory results. Akin et al. conducted a statistical analysis of 2689 consecutive patients from the European Registry for Patients with Mechanical Circulatory Support undergoing LVAD implantation. The univariate analysis revealed that the female gender is one of the independent mortality predictors.^24^

LVAD implantation in patients presenting with end-stage HF is known to be associated with a high risk of acute kidney injury (AKI) following renal replacement therapy (RRT).^25^ Both male and female patients in out cohort presented with initially impaired kidney function and elevated creatinine rates. Patients with preoperative chronic impairment of kidney function undergoing LVAD therapy are at an even greater risk of postoperative RRT or permanent dialysis.^26^ Although, our cohort illustrated no significant difference in kidney function between male and female groups, female patients presented with a significantly higher rate of postoperative RRT. Alba et al. researched the predictive factors for AKI after LVAD implantation and demonstrated that longer time on cardiopulmonary bypass, higher intraoperative blood loss, and reoperation were associated with a higher rate of AKI. Unfortunately, the female gender was not separately analyzed.

This study does not demonstrate an increase in in-hospital and follow-up mortality following LVAD implantation in female patients compared to the matched male individuals. Moreover, the regression analysis showed that the female gender is not an independent predictor of in-hospital and follow-up mortality. However, female gender is associated with higher rates of LVAD-thrombosis and higher risk of acute kidney injury with a long-term dialysis.

### Study limitations

The retrospective nonrandomized nature of the study coming from a single center with a limited number of patients may have an impact on the outcomes and the study power, and can leave room for bias.

## Conclusions

Durable continuous flow left ventricular assist devices as bridge to transplantation or recovery in female patients are associated with a higher risk of acute kidney injury requiring RRT and higher risk of LVAD-thrombosis. Nevertheless, survival rates between genders are similar. Further studies on the gender specific therapy protocols in patients with end-stage heart failure requiring durable LVAD are necessary to explore gender specific risks capable of affecting the outcomes of this high-risk patient group.
